# A scDb-based trivalent bispecific antibody for T-cell-mediated killing of HER3-expressing cancer cells

**DOI:** 10.1038/s41598-021-93351-0

**Published:** 2021-07-06

**Authors:** Nadine Aschmoneit, Sophia Steinlein, Lennart Kühl, Oliver Seifert, Roland E. Kontermann

**Affiliations:** 1 Institute of Cell Biology and Immunology, University of Stuttgart, Allmandring 31, 70569 Stuttgart, Germany; 2 Stuttgart Research Center Systems Biology (SRCSB), University of Stuttgart, 70569 Stuttgart, Germany

**Keywords:** Protein design, Antibody therapy

## Abstract

HER3 is a member of the EGF receptor family and elevated expression is associated with cancer progression and therapy resistance. HER3-specific T-cell engagers might be a suitable treatment option to circumvent the limited efficacy observed for HER3-blocking antibodies in clinical trials. In this study, we developed bispecific antibodies for T-cell retargeting to HER3-expressing tumor cells, utilizing either a single-chain diabody format (scDb) with one binding site for HER3 and one for CD3 on T-cells or a trivalent bispecific scDb-scFv fusion protein exhibiting an additional binding site for HER3. The scDb-scFv showed increased binding to HER3-expressing cancer cell lines compared to the scDb and consequently more effective T-cell activation and T-cell proliferation. Furthermore, the bivalent binding mode of the scDb-scFv for HER3 translated into more potent T-cell mediated cancer cell killing, and allowed to discriminate between moderate and low HER3-expressing target cells. Thus, our study demonstrated the applicability of HER3 for T-cell retargeting with bispecific antibodies, even at moderate expression levels, and the increased potency of an avidity-mediated specificity gain, potentially resulting in a wider safety window of bispecific T-cell engaging antibodies targeting HER3.

## Introduction

Elevated expression of HER3, a member of the EGF receptor family, has been reported to play an essential role in cancer progression and correlates with worse overall survival in many solid tumors^1,2^. Additionally, a number of studies revealed the upregulation of HER3 as an important resistance mechanism upon EGFR and HER2-targeted therapy^3–5^, emphasizing the importance in developing novel therapeutic strategies targeting HER3. HER3 comprises an impaired tyrosine kinase domain and requires heterodimerization with and transphosphorylation by other members of the EGFR family for activation and signaling^1^. Consequently, antibodies have been developed to interfere with ligand binding and/or receptor dimerization^6^. Currently, there are more than two dozen antibodies investigated in preclinical studies^7,8^. However, there is still no approved treatment targeting HER3 and clinical trials for the two most prominent candidates patritumab^9^ and seribantumab^10^ were terminated due to lack of efficacy (NCT02134015, NCT03241810). As extensive signaling crosstalk and redundancy was observed between the EGFR family members, combinatorial treatment strategies have been developed^11^, including antibody combinations and bispecific antibodies for dual targeting of HER3 and other members of the EGFR family^12–17^. However, also these approaches face several limitations. For example, results from a phase II study of a bispecific antibody, duligotuzumab, targeting EGFR and HER3, in K-RAS wild-type metastatic colorectal cancer patients could not demonstrate an improved therapeutic activity in combination with chemotherapy compared to Cetuximab plus chemotherapy^18^. This has prompted the development of alternative approaches, such as HER3-directed antibody–drug conjugates (ADCs)^19–21^, i.e. using HER3 as a target structure and uncoupling therapeutic activity from receptor signaling.

Bispecific antibodies cross-linking tumor cells and T-cells, independently of binding the MHC through the TCR, represent a rapidly expanding treatment modality developed for cancer therapies^22,23^. Simultaneous binding of a tumor-associated antigen (TAA) and the CD3ε chain of the T-cell receptor (TCR)/CD3 complex leads to the close apposition of target and effector cell and thereby the activation of the T-cell. Secretion of cytokines and cytotoxic effector proteins by the T-cell eventually results in killing of the targeted tumor cell. One major obstacle of this concept is the extraordinary potency of T-cell responses which can potentially lead to the attack of non-tumor cells with low expression level of the TAA and/or systemic cytokine-associated adverse events^22^. Especially bispecific antibodies comprising an Fc part have been found to induce an Fc-mediated immune cell activation eventually resulting in a cytokine storm^24,25^. These effects can be avoided by using Fc-less bispecific antibodies such as the bispecific T-cell engager BiTE^26^, the dual-affinity-re-targeting DART^27^ or single-chain diabodies (scDb)^28^. These formats use molecular designs creating a 1:1 valency for CD3 and the TAA. Due to their small size and short distance between the two binding sites they can mediate tight contacts between target cell and T-cells, and thus efficient T-cell activation. The potentials of bispecific T-cell engagers to treat hematologic malignancies are well established, with blinatumomab, which is a bispecific BiTE directed against CD19 and CD3, approved for the treatment of acute lymphoblastic leukemia^29^. However, their application for the treatment of solid tumors face several challenges, including adverse events due to on-target off-tumor toxicities^22^. Recent studies demonstrated that novel bispecific antibody formats with a 2 + 1 stoichiometry can result in an avidity-mediated specificity gain through bivalent binding to the TAA^30–32^, while monovalent binding to the trigger molecule CD3 on T-cells avoids unspecific or non-targeted CD3-crosslinking and T-cell activation^23,33–35^.

In the present study, we used a HER3-specific human antibody (3–43)^36^ to generate bispecific T-cell engagers based on the scDb format^37^. To employ avidity effects in target cell binding, we further modified this bivalent, bispecific HER3xCD3 scDb format to obtain a trivalent, bispecific molecule by fusing an additional anti-HER3 scFv to the C-terminus of the scDb moiety (scDb-scFv). Thus, this trivalent scDb-scFv combines bivalent binding to the TAA HER3 and monovalent binding to CD3, positioning the HER3 and the CD3 binding moieties on opposite sides of the molecule. We analyzed the effects on target cell binding, T-cell activation and T-cell mediated target cell killing in vitro, demonstrating that these HER3-targeting T-cell engagers efficiently mediate target cell destruction, with a superior activity observed for the trivalent, bispecific scDb-scFv.

## Results

### Generation of bispecific antibodies

**Figure 1:**
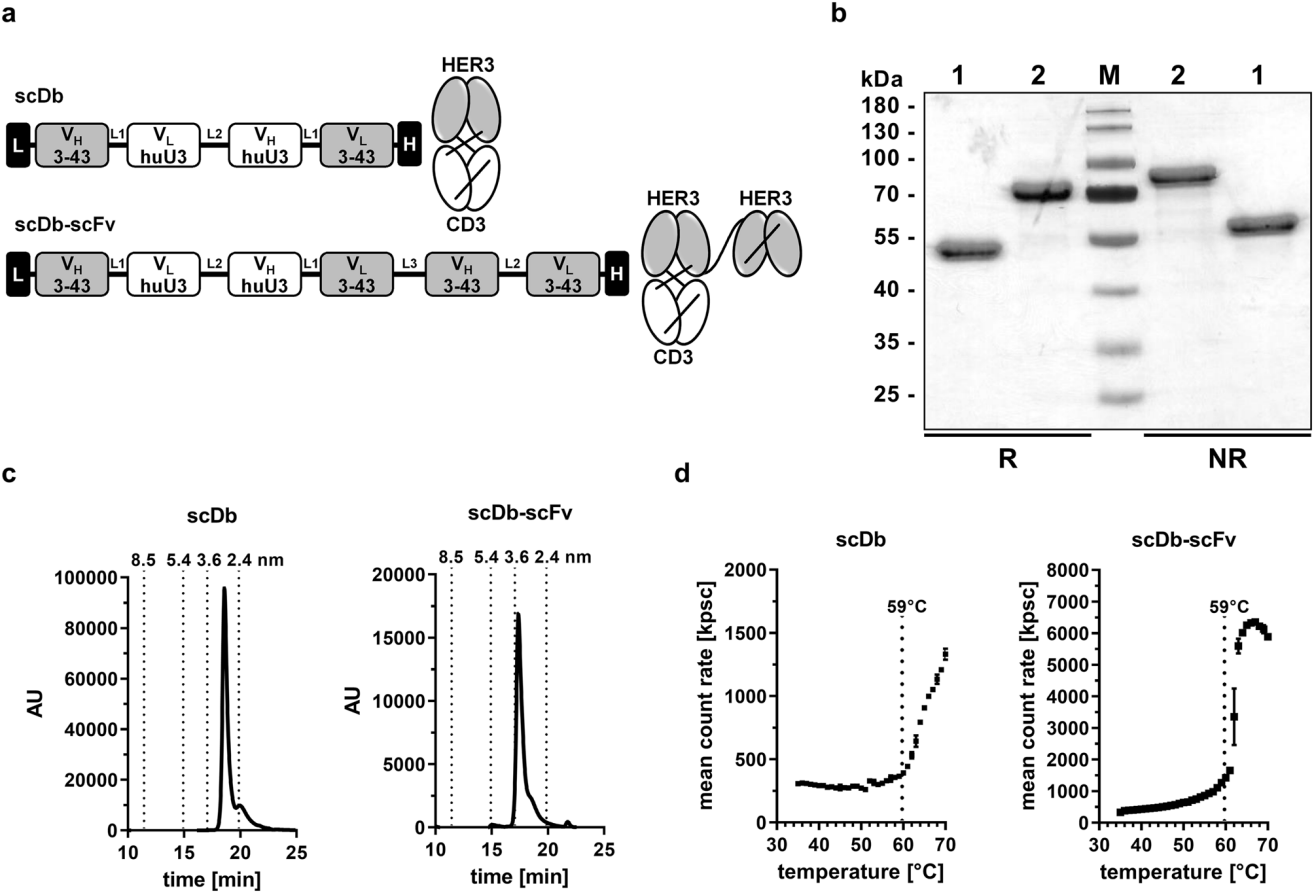
Biochemical characterization of scDb and scDb-scFv. (**a**) Composition and schematic illustration of scDb (1) and scDb-scFv (2). L, Igκ chain leader sequence. L1, G_4_S. L2, (G_4_S)_3_. L3, AAAGGS(G_4_S)GGGT, H, hexahistidyl-tag. (**b**) SDS PAGE analysis (12% PAA, 2 µg/lane, Coomassie blue staining) of (1) scDb and (2) scDb-scFv under reducing (R) and non-reducing (NR) condition. M, protein marker. (**c**) Size-exclusion chromatography by HPLC using a Tosoh TSKgel SuperSW mAb HR column. (**d**) Thermo stability determined by dynamic light scattering using the ZetaSizer Nano ZS (Malvern). Mean ± SD, n = 3.

A bivalent bispecific scDb binding to HER3 and CD3 was generated by combining the antigen-binding site of the anti-HER3 antibody 3–43 with the antigen-binding site of a humanized anti-CD3 antibody (huU3) derived from UCHT1. Furthermore, a trivalent bispecific scDb-scFv molecule was generated by fusing an additional anti-HER3 scFv to the C-terminus of the anti-HER3xCD3 bispecific scDb (Fig. 1a and Supplementary Figure 1). Both, the scDb and the scDb-scFv were produced in transiently transfected HEK293-6E cells and purified by immobilized metal ion affinity chromatography (IMAC) followed by a preparative size-exclusion chromatography step (SEC). Protein purity was confirmed by SDS-PAGE analysis under reducing and non-reducing conditions. The determined apparent molecular masses for scDb (~ 52 kDa) and scDb-scFv (~ 72 kDa) correlated to their calculated molecular masses of 55 kDa and 83 kDa, respectively (Fig. 1b). In analytical size-exclusion chromatography (SEC), both scDb and scDb-scFv eluted as one major peak. A Stokes radius of 2.8 nm was determined for the scDb and an increased size of 3.4 nm was measured for scDb-scFv (Fig. 1c). Dynamic light scattering revealed an aggregation temperature (Tm) of approximately 59 °C for both proteins (Fig. 1d).

### Superior binding of the scDb-scFv to HER3-expressing target cells

**Figure 2:**
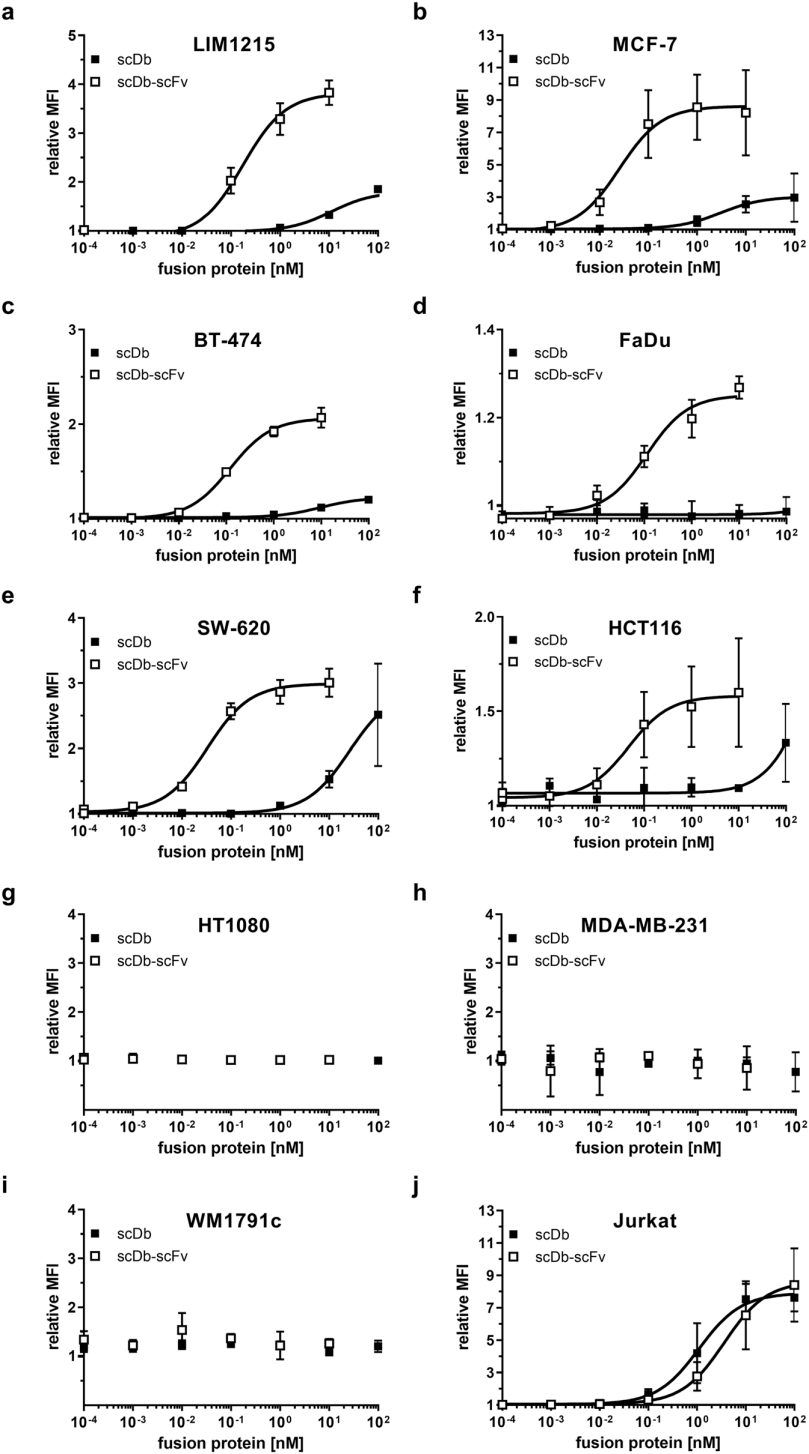
Binding properties of scDb and scDb-scFv. Binding to (**a**) LIM1215, (**b**) MCF-7, (**c**) BT-474, (**d**) FaDu, (**e**) SW-620, (**f**) HCT116, (**g**) HT1080, (**h**) MDA-MB-231, (**i**) WM1791c and (**j**) CD3-expressing Jurkat cells was analyzed in flow cytometry. Bound protein was detected using a PE-labeled anti-His mAb. Mean ± SD, n = 3.

Tumor cell binding was analyzed by flow cytometry using a panel of different cell lines expressing various HER3 receptor levels. Binding analysis on the colon carcinoma cell line LIM1215 (~ 20,000 HER3/cell), HCT116 (< 1900 (1520) HER3/cell) and SW-620 (< 1900 (1160) HER3/cell), the breast cancer cell lines MCF-7 (~ 18,000 HER3/cell), BT-474 (~ 11,000 HER3/cell) and the pharynx carcinoma cell line FaDu (~ 3000 HER3/cell) revealed superior binding for the scDb-scFv compared to scDb, due to the avidity effect of this molecule. EC_50_ values for the scDb-scFv were in the subnanomolar range (30–200 pM), whereas the scDb showed up 1000-fold weaker binding (Fig. 2a–f). On the low HER3-expressing cell line FaDu binding of scDb was basically neglectable, while scDb-scFv showed binding with an EC_50_ value similar to that observed for the other tested cell lines. On the HER3-negative cell lines HT1080 (fibrosarcoma), MDA-MB-231 (breast adenocarcinoma) and WM1791c (melanoma) no binding was detected for scDb or scDb-scFv (Fig. 2g–i). On the CD3-expressing cell line Jurkat, scDb-scFv and scDb showed similar binding with EC_50_ values of 4.0 ± 1.3 nM and 1.6 ± 1.3 nM, respectively, in line with monovalent CD3 binding of both molecules (Fig. 2j) (Table 1).

**Table 1:** Binding to HER3-expressing tumor cells of scDb and scDb-scFv. EC_50_ [pM], Mean ± SD, n = 3. ^**#**^ For the HER3 low-expressing cell lines SW-620 and HCT116 the extrapolated values under the detection limit of 1900 molecules/cell (Qifikit) are depicted. *p < 0.05; **p < 0.01; n.s. p > 0.05; n.d., not determined.

Cell line	HER3 expression	scDb-scFv	scDb	p value
LIM1215	20,000 HER3/cell	200 ± 100	11,700 ± 6000	*
MCF-7	18,000 HER3/cell	30 ± 20	4700 ± 6200	*
BT-474	11,000 HER3/cell	200 ± 170	9700 ± 2200	**
FaDu	3000 HER3/cell	100 ± 30	–	n.d.
SW-620^#^	1160 HER3/cell	30 ± 3	30,800 ± 30,200	**
HCT116^#^	1520 HER3/cell	50 ± 10	–	n.d.
HT1080	HER3^−^	–	–	n.d.
MDA-MB-231	HER3^−^	–	–	n.d.
WM1791c	HER3^−^	–	–	n.d.

### Activation of effector T-cells by scDb-scFv

**Figure 3:**
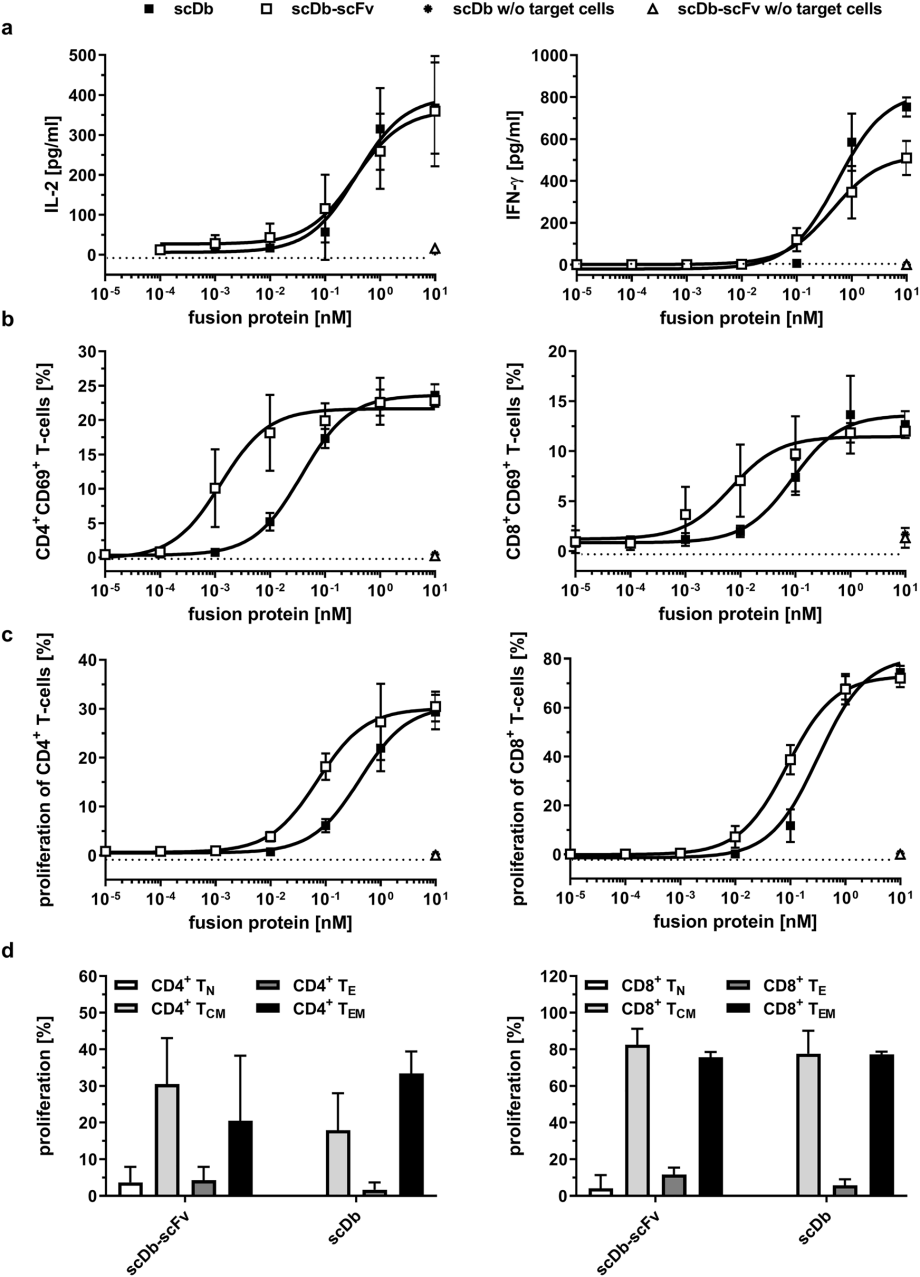
Activity of scDb and scDb-scFv on cytokine release, T-cell activation and proliferation. (**a**) IL-2 and IFN-γ release and (**b**) CD69 expression of CD4^+^ and CD8^+^ T-cells mediated by scDb and scDb-scFv. PBMCs were co-cultured with MCF-7 cells in presence of fusion protein. Cytokine release was determined after 24 h (IL-2) or 48 h (IFN-γ) using sandwich ELISA. CD69 expression on T-cells was analyzed after 24 h in flow cytometry. (**c**) Proliferation of CD4^+^ and CD8^+^ T-cells was measured by CFSE dilution in flow cytometry. (**d**) Proliferation of naïve (T_N_, CD45RA^+^, CCR7^+^), central memory (T_CM_, CD45RA^−^, CCR7^+^), effector (T_E_, CD45RA^+^, CCR7^−^) and effector memory (T_EM_, CD45RA^−^, CCR7^−^) subpopulations of CD4^+^ T-cells and CD8^+^ T-cells was determined by CFSE dilution in flow cytometry. Mean ± SD of three independent experiments (n = 3) using PBMCs from one donor for each analysis. In total, PBMCs from 4 different donors were used.

Simultaneous binding of the scDb and the scDb-scFv to tumor and effector cells and subsequent T-cell activation was investigated in co-culture assays. First, IL-2 and IFN-γ release was determined. Both, scDb-scFv and scDb showed a concentration-dependent cytokine release by T-cells after 1 or 2 days of incubation, respectively (Fig. 3a). Of note, no significant difference in IL-2 or IFN-γ release was observed for the scDb and the scDb-scFv. Importantly, neither scDb-scFv nor scDb activated T-cells in terms of cytokine release in the absence of target cells (Fig. 3a). Next, we investigated early activation of T-cells by CD69-expression. After one day, for both scDb-scFv and scDb a dose-dependent activation of CD4^+^ and CD8^+^ T-cells was observed, reaching a similar percentage of CD69^+^ T-cells. However, scDb-scFv showed a ~ 30-fold (CD4^+^ T-cells) and ~ eightfold (CD8^+^ T-cells) lower EC_50_ value compared to the scDb (Fig. 3b). Again, no activation of T-cells in the absence of target cells was observed. Additionally, the effect of scDb-scFv and scDb on T-cell proliferation was investigated. For CD8^+^ T-cell proliferation, scDb-scFv showed a threefold stronger activity (EC_50_: 90 ± 30 pM) compared to the scDb (EC_50_: 300 ± 70 pM) and a sixfold stronger activity in inducing proliferation of CD4^+^ T-cells with an EC_50_ value of 80 ± 20 pM for the scDb-scFv compared to an EC_50_ value of 500 ± 100 pM for the bivalent scDb (Fig. 3c). Concerning the activation of T-cell subpopulations, no difference between scDb-scFv and scDb was observed. Treatment with both proteins mainly led to proliferation of central memory (T_CM_) and effector memory (T_EM_) CD4^+^ and CD8^+^ T-cells (Fig. 3d). Thus, compared to scDb the scDb-scFv molecule is more potent in mediating activation and proliferation of CD4^+^ and CD8^+^ T-cells while induction of cytokine release is not increased (Table 2).

**Table 2:** T-cell activation of scDb and scDb-scFv. EC_50_ [pM], Mean ± SD, n = 3. *p < 0.05; **p < 0.01; n.s. p > 0.05.

	scDb-scFv	scDb	p value
IFN-γ	570 ± 460	610 ± 230	n.s.
IL-2	400 ± 230	400 ± 150	n.s.
CD4^+^CD69^+^	3 ± 3	100 ± 100	**
CD8^+^CD69^+^	20 ± 20	160 ± 120	n.s.
Proliferation CD4^+^ T cells	80 ± 20	500 ± 100	**
Proliferation CD8^+^ T cells	90 ± 30	300 ± 70	**

### Redirected lysis of HER3-positive cancer cell lines by scDb-scFv

**Figure 4:**
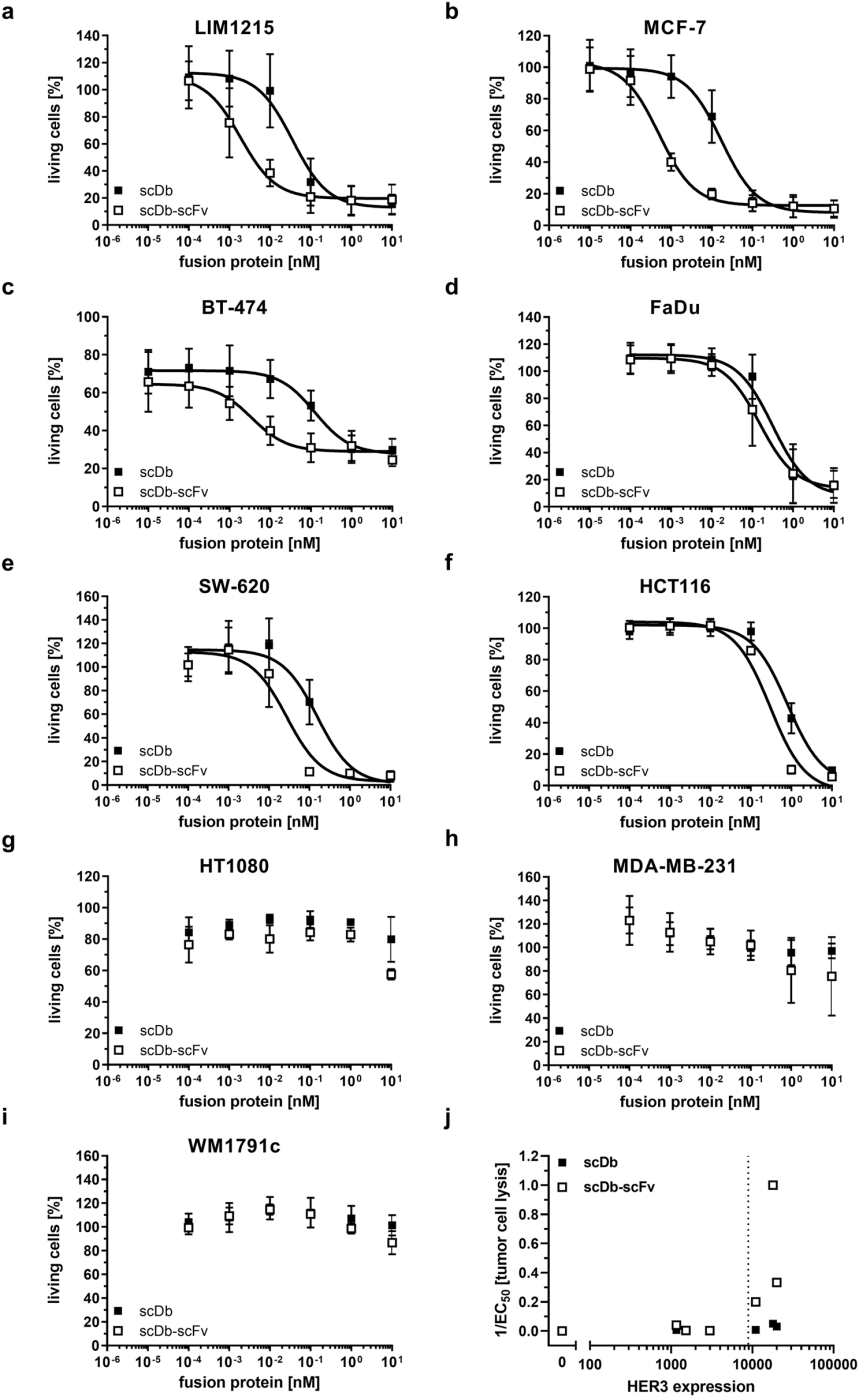
Effect of scDb and scDb-scFv on cytotoxic potential of PBMCs. Target cells ((**a**) LIM1215, (**b**) MCF-7, (**c**) BT-474, (**d**) FaDu, (**e**) SW-620, (**f**) HCT116, (**g**) HT1080, (**h**) MDA-MB-231 or (**i**) WM1791c cells) were incubated with a serial dilution of scDb or scDb-scFv and PBMCs in an effector:target cell ratio (E:T) of 10:1. After 3 days, cell viability was determined using crystal violet staining. Mean ± SD. On the low expressing cell lines SW-620, HCT116, HT1080, MDA-MB-231 and WM1791c three independent experiments (n = 3) were performed using PBMCs from one donor. On the cell lines LIM1215, MCF-7, BT-474 and FaDu three independent experiments for each donor using PBMCs from two different donors were performed (n = 6). In total, PBMCs from 5 different donors were used. (**j**) Potency of scDb and scDb-scFv versus HER3 expression.

HER3-positive cell lines with medium (LIM1215: 20,000 HER3/cell; MCF-7: 18,000 HER3/cell; BT-474: 11,000 HER3/cell), low (FaDu: 3000 HER3/cell; SW-620: < 1900 (1160) HER3/cell; HCT116: < 1900 (1520) HER3/cell and no detectable antigen expression (HT1080, MDA-MB-231 and WM1791c) were used to determine the cytotoxic effects of PBMCs on target cells mediated by scDb-scFv and scDb. Both proteins were able to redirect unstimulated PBMCs to lyse HER3-expressing target cells in a concentration-dependent manner. For the HER3-expressing cell lines, no significant difference between the two proteins was observed regarding efficacy (maximum target cell lysis at 10 nM bispecific antibody) (Fig. 4a–f). Thus, treatment with scDb-scFv and scDb led to 80–90% killing of LIM1215, MCF-7, FaDu, SW-620 and HCT116 cells and 60–70% of BT-474 cells were lysed upon treatment with scDb-scFv or scDb. In contrast, tremendous differences were observed regarding the potency (EC_50_ value in cell killing) (Table 3). Superior effects on target cell killing were observed for the scDb-scFv compared to scDb especially on target cells with medium HER3 expression levels (LIM1215: 18-fold, MCF-7: 32-fold, BT-474: 34-fold) (Fig. 4a–c). Of note, only a two- to sixfold higher potency for the scDb-scFv compared to the scDb was observed on the low HER3-expressing cell lines FaDu, SW-620 and HCT116 (Fig. 4d–f). On the HER3-negative cell lines HT1080, MDA-MB-231 and WM1791c only a slight cytotoxic effect of the scDb-scFv at highest concentrations was observed (Fig. 4g–i). Plotting 1/EC_50_ values versus HER3 expression revealed a strong increase in potency for the scDb-scFv molecule above approximately 9000 HER3 molecules/cell, which was not seen for scDb, indicating an avidity-mediated gain in target cell killing for cancer cells expressing medium levels of HER3 (Fig. 4j).

**Table 3:** T-cell mediated killing of tumor cells using scDb and scDb-scFv. EC_50_ [pM], Mean ± SD, n = 2 with PBMCs from two donors for LIM1215, MCF-7, BT-474 and FaDu cells; n = 3 with PBMCs from one donor for SW-620, HCT116, HT1080, MDA-MB-231 and WM1791c cells. ^**#**^For the HER3 low-expressing cell lines SW-620 and HCT116 the extrapolated values under the detection limit of 1900 molecules/cell (Qifikit) are depicted. *p < 0.05; **p < 0.01; ****p < 0.0001; n.s. p > 0.05; n.d., not determined.

Cell line	HER3 expression	scDb-scFv	scDb	Ratio of EC_50_	p value
LIM1215	20,000 HER3/cell	3 ± 3	40 ± 22	11	**
MCF-7	18,000 HER3/cell	1 ± 0.2	19 ± 6	31	****
BT-474	11,000 HER3/cell	5 ± 5	128 ± 37	29	****
FaDu	3000 HER3/cell	464 ± 719	724 ± 911	2	n.s.
SW-620^#^	1160 HER3/cell	26 ± 10	150 ± 50	6	**
HCT116^#^	1520 HER3/cell	300 ± 30	800 ± 300	3	*
HT1080	HER3^−^	–	–	–	n.d.
MDA-MB-231	HER3^−^	–	–	–	n.d.
WM1791c	HER3^−^	–	–	–	n.d.

## Discussion

In this study, we report the generation of HER3-targeting bispecific, bivalent or trivalent antibody molecules capable of redirecting T-cells to HER3-expressing cancer cells leading to efficient killing of target cells. The scDb-based trivalent, bispecific T-cell engaging antibody utilizing a 2 + 1 stoichiometry (scDb-scFv) demonstrated enhanced T-cell activation and higher potency in T-cell mediated target cell killing compared to the bivalent bispecific scDb. Fusion of an additional binding moiety for the TAA to the scDb increased the binding to HER3 on target cells. This can be attributed to avidity effects, in accordance with data obtained for the bivalent IgG 3–43 compared to scFv 3–43^36^. IgG 3–43 binds monovalently with an affinity of 11 nM to HER3 as determined by QCM measurements, while IgG 3–43 exhibits a strongly increased binding to target cells, with EC_50_ values in the low picomolar range^36^.

The correlation between antigen density and potency is of vital importance for safety and efficacy, as it directly affects the number of contact points between target cell and T-cell^38^. For a BiTE targeting ephrin type-A receptor 2 (EpAh2), a trend in which the potency of bscEphA2xCD3 increased as the number of EphA2 binding sites on the tumor cells increased was observed^39^. Similarly, a bispecific antibody against HER2 and CD3 showed high potency towards cells with amplified or overexpressed HER2 (~ 2 × 10^6^ molecules/cell) and ~ 40-fold reduced potency for cells with lower target expression (~ 15,000 molecules/cell)^40^. In line with this, scDb-scFv showed an approximately 100-fold reduced T-cell mediated killing of low HER3-expressing tumor cells (FaDu: ~ 3000 HER3/cell) compared to intermediate HER3-expressing tumor cells (MCF-7: ~ 18,000 HER3/cell).

Comparing the potency of scDb and scDb-scFv, a more pronounced avidity effect of the scDb-scFv on medium compared to low HER3-expressing cell lines was observed. Presumably, some of the scDb-scFv molecules only bind monovalenty to the HER3 low-expressing target cells, while mostly bivalent binding of the scDb-scFv to HER3 is observed on HER3-intermediate expressing cells. Similarly, For HER2-targeting 2 + 1 T-cell engagers is was shown that T-cell-dependent bispecific antibody-mediated killing is dependent on target expression^41^. A similar dependency between mono- and bivalent binding and the target expression was observed by Zuckier and coworkers, where the Ars binding IgG1 monoclonal antibody mAb 36–65 showed increased binding in presence of a high density of the antigen du to simultaneous binding of both arms of the IgG compared to a monovalent interaction of only one Fab arm with the antigen if the antigen is only present in a low density^42^.

Importantly, while early T-cell activation, proliferation and target cell killing was strongly improved for scDb-scFv, cytokine release was not affected. There is growing evidence that the cytotoxic activity is uncoupled from cytokine release, defined by two activation thresholds, and that cytokine release is dispensable for target cell killing^43^. Thus, a low number of TCR:peptide-MHC complexes is sufficient to trigger T-cell-mediated lysis, whereas a higher number of complexes are required for the secretion of cytokines. This may be related to the separate but intertwined signaling pathways of the TCR differentially regulating release of cytokines and lytic effector molecules^44–46^. This is supported by findings that in response to bispecific T-cell engagers, T-cells retained their cytolytic activity despite a lack of cytokine release^47^ and that anti-CD3 antibodies have been identified that, when used for T-cell retargeting, can mediate robust target cell killing while stimulating only very low levels of cytokine release.

Deploying avidity effects by introducing an additional binding site for HER3, resulted in an effective T-cell engaging bispecific antibody (scDb-scFv) in the 2 + 1 stoichiometry, differentiating cells of high and low HER3 expression. The increased avidity potentially improves tumor selectivity and consequently may lower the risk of on-target off-tumor adverse effects^32^. For a T-cell dependent bispecific antibody targeting HER2 and CD3 it has been shown that introducing an additional binding moiety for HER2 led to increased tumor cell selectivity and highly potent targeting of HER2-positive target cells, while sparing cells that express low amounts of HER2^41^. In line with this, Bacac et al. demonstrated that a bivalent binding mode for the TAA CEA translated into selective killing of high CEA-expressing tumor cells while sparing normal epithelial cells resulting in a wide safety window for their CEA TCB^31^. Additionally, for a divalent (scFv’)_2_ a correlation between valency and retention time at the tumor was described, providing further evidence that the valency directly affects tumor localization^48^. However, further studies are required to investigate the safety profile of HER3-targeting bispecific antibodies.

The close proximity of target and effector cell binding moieties due to the small, compact and rigid scDb format potentially facilitates the formation of a tight immune synapse similar to the BiTE format^49–51^. This property is maintained in the scDb-scFv with the scFv moiety fused to the scDb through a short linker providing sufficient flexibility to allow dual binding to HER3 and efficient engagement of T-cells. However, it has also been shown that the location and distance of the targeted epitope from the target cell membrane affects formation of the immune synapse^38^. Targeting membrane-proximal epitopes reportedly results in the exclusion of the negative regulatory protein CD45 immune synapse^52^ translating into increased activation of T-cells and more potent T-cell mediated target cell killing^51–55^. Accordingly, the high potency of scDb-scFv and scDb might not only be attributed to the format and the resulting avidity-mediated specificity gain but also to the chosen anti-HER3 antibody binding to the membrane-proximal domains III and IV of HER3^36^.

Furthermore, the epitope and the affinity of the CD3 binding site have been shown to affect efficacy and safety of bispecific T-cell engagers^56^, thus providing a rational for further improvement of the safety properties of HER3-targeting T-cell engagers. Especially in the context of solid tumors, redirecting CD3^+^ T-cells has been reported to be associated with a number of challenges probably limiting or reducing the anti-tumor efficacy, including the recruitment of counterproductive CD3^+^ T-cell subsets, dose limiting cytokine storm, the presence of an immunosuppressive tumor microenvironment and on-target and off-tumor binding^22,57^. Several disadvantages are associated with polyclonal T-cell activation, including the recruitment of naïve, exhausted or regulatory T-cells^58,59^. The subset of memory T-cells can be further divided into central memory T-cells mostly trafficking to lymphoid tissue and exhibiting a high proliferative capacity^60^ and effector memory T-cells localizing in peripheral tissue with rapid development of effector functions^61^. In the present study, both scDb and scDb-scFv mostly activated CD8^+^ central memory (CD45RA^−^CCR7^+^) and effector memory (CD45RA^−^CCR7^−^) T-cells while no proliferation of naïve CD8^+^ T-cells was observed. Importantly, it has been shown that the tissue-resident memory T-cell phenotype (CD8^+^CD69^+^CD103^+^) expresses high levels of cytotoxic molecules and is associated with a good clinical outcome in cancer^62^.

The main challenge in therapeutic approaches targeting HER3 is the low expression in tumors (usually below 50,000 receptors/cell)^63^ together with expression in normal tissue^64^. Past studies involving monoclonal antibodies targeting HER3 have shown manageable side effects. Lumretuzumab, a humanized anti-HER3 IgG1 monoclonal antibody, was well tolerated in a phase I clinical trial^65^ and the toxicity profile of Lumretuzumab in combination with the EGFR-blocking agents Erlotinib and Cetuximab in a Phase Ib study was manageable with gastrointestinal and skin toxicities^66^. Similarly, a phase II trial of Exemestane plus Seribantumab (anti-HER3 mAb) revealed diarrhea and nausea as the most common adverse events^67^. Avidity-mediated discrimination between moderate and low HER3-expressing cells in combination with monovalent binding to the trigger molecule CD3 potentially lowers the risk of on-target off-tumor adverse effects which might lead to a preferable therapeutic window.

In summary, our data demonstrate that scDb-scFv is a potent format for the generation of T-cell engaging antibodies. Monovalent binding to the trigger molecule CD3 on T-cells and bivalent binding to the membrane-proximal tumor antigen HER3 enabled effective formation of immune synapses and triggering T-cell mediated tumor cell lysis, emphasizing the suitability of HER3 as a tumor target for redirecting T-cells to tumor cells discriminating between moderate and low HER3-expressing target cells. How this translates into potent antitumor activities while maintaining a favorable safety profile demands further in vivo studies.

## Material and methods

### Materials

Antibodies were purchased from Biolegends (PerCP/Cy5.5 anti-human CD3, 317336; PE anti-human CCR7, 353204; APC anti-human CD45RA; 304112; PE anti-human CD69, 310906) or Miltenyi Biotec (anti-human CD4-VioBlue, 130-097-333; anti-human CD8-PE/Vio770, 130-096-556; anti-His-PE, 130-092-691). Human IFN-γ DuoSet ELISA kit (DY285) and Human IL-2 DuoSet ELISA kit (DY202) were obtained from R&D Systems. CellTrace CFSE Cell Proliferation Kit (C34554) was purchased from Thermo Fisher Scientific. FaDu, BT-474, HCT116, HT1080, WM1791c and MCF-7 cells were obtained from different sources and cultured as described previously^36,68–70^. LIM1215 were obtained from Merck KGaA (10092301-1VL), SW-620 cells and MDA-MB-231 cells were obtained from CLS Cell Lines Service GmbH (300466), respectively, and cultured in RPMI-1640 (Thermo Fisher Scientific, 11875), 10% FBS (Pan Biotech, P30-3309). The cell line WM1791c was purchased as authenticated STR-profiled stock directly from the vendor (Wistar; WC00086). All other cell lines were authenticated by SNP profiling (Multiplexion GmbH, Friedrichshafen, Germany). Human peripheral blood mononuclear cells (PBMCs) were isolated from buffy coats of healthy donors (Blood bank, Klinikum Stuttgart) by density gradient centrifugation (Lymphocyte Seperation Medium 1077, Promocell, C-44010) and cultivated in RPMI-1640, 10% FBS. PBMCs preparations from 9 different donors were prepared and used for the experiments.

### Antibody production and purification

To generate the scDb, variable domains of the heavy and light chains of the 3–43^36^ and a humanized version of UCHT1 (huU3) were cloned into the pSecTagAL1 vector [a modified version of pSecTagA (Invitrogen, Thermo Fisher Scientific, V90020)]. Connecting an additional scFv3-43 to the scDb using a (G_4_S)_3_ linker resulted in the scDb-scFv (WO2018050848A1). All proteins were produced in transiently transfected HEK293-6E cells (NRC Biotechnology Research Institute, Canada) using polyethylenimine (PEI; linear, 25 kDa, Sigma-Aldrich, 764604). Supernatants were harvested 96 h post transfection, proteins were precipitated by addition of 390 g/l (NH_4_)_2_SO_4_, purified by immobilized metal ion affinity chromatography (IMAC) followed by size-exclusion FPLC on a Superdex 200 10/300 GL column (PBS as mobile phase, 0.5 ml/min flow rate).

### Biochemical characterization

Purified proteins were analyzed by SDS-PAGE under reducing and non-reducing conditions and stained with Coomassie Brilliant Blue G-250. Purity and integrity of the proteins were determined by size-exclusion chromatography using a Waters 2695 HPLC and a TSKgel SuperSW mAb HR column (Tosoh Bioscience) at a flow rate of 0.5 ml/min with 0.1 M Na_2_HPO_4_/NaH_2_PO_4_, 0.1 M Na_2_SO_4_, pH 6.7 as mobile phase. Thyroglobulin (669 kDa, Sr 8.5 nm), β-Amylase (200 kDa, Sr 5.4 nm), bovine serum albumin (67 kDa, Sr 3.55 nm) and carbonic anhydrase (29 kDa, Sr 2.35 nm) were used as reference proteins.

### Thermal stability

ZetaSizer Nano ZS (Malvern) was used to analyze the thermal stability of the proteins by dynamic light scattering. Purified protein was exposed to increasing temperature (30–70 °C) in 1 °C intervals with 2-min equilibration steps. The aggregation point was defined by the starting point of the increase in the mean count rate.

### Cell binding

1 × 10^5^ target cells were incubated with the recombinant proteins for 1 h at 4 °C. Bound protein was detected using a PE-conjugated anti-hexahistidyl tag mAb (Miltenyi Biotec). Incubation and washing steps were performed in PBS, 2% FBS, and 0.02% sodium azide. Fluorescence was measured by MACSQuant VYB (Miltenyi Biotec) and data were analyzed using FlowJo (Tree Star). Relative median fluorescence intensities (MFI) were calculated as followed: relative MFI = ((MFIsample − (MFIdetection − MFIcells))/MFIcells).

### Early activation of T cells

2 × 10^4^ MCF-7 cells/well seeded the day before were incubated with fusion proteins for 15 min followed by the addition of 2 × 10^5^ PBMCs/well. Early activation was determined by CD69 expression. Therefore, PBMCs were harvested after 24 h and CD69 expressing CD4^+^ and CD8^+^ T cells were identified by flow cytometry using MACSQuant Analyzer 10 (Miltenyi Biotec).

### IL-2/IFN-γ release assay

2 × 10^4^ MCF-7 cells/well were incubated with fusion protein for 15 min at RT followed by addition of 2 × 10^5^ PBMCs/well. After 24 h (IL-2) or 48 h (IFN-γ) cell-free supernatants of the coculture were harvested and IL2/IFN-γ concentration was determined using sandwich ELISA.

### T cell proliferation

To analyze the proliferative effect on T cells, PBMCs were labeled with carboxyfluorescein diacetate succinimidyl ester (CFSE). Therefore, PBMCs were resuspended in PBS with 0.1% BSA at a cell count 1 × 10^6^ cells/ml and CFSE with a final concentration of 625 nM was added. After incubation at 37 °C for 15 min, internalization of CFSE was stopped by incubation on ice for 5 min and addition of double the volume of RPMI 1640 + 10% FBS. 2 × 10^4^ MCF-7 cells/well were incubated with fusion protein for 15 min at RT followed by addition of 2 × 10^5^ CFSE-labeled PBMCs/well. After 6 days, immune cells of interest were labeled with fluorescence-conjugated antibodies directed against respective cell-surface markers and proliferation was measured by multicolor flow cytometry analysis using MACSQuant Analyzer 10 (Miltenyi Biotec).

### Cytotoxicity

Previously seeded target cells (2 × 10^4^ cells/well) were incubated with fusion proteins for 15 min at RT prior to addition of PBMCs (E:T ratios of 10:1). After 3 days, supernatants were discarded and viable target cells were stained with crystal violet. Staining was solved in methanol (50 µl/well) and optical density measured at 550 nm using the Tecan spark (Tecan).

### Statistics

All data are represented as mean ± SD. Significances were calculated by GraphPad Prism 7.0 and results were compared by *t* test.

## Supplementary Information

Supplementary Figure 1.

## Data Availability

The datasets generated during and/or analyzed during the current study are available from the corresponding author on reasonable request.
