# Supplementary material for: A scDb-based trivalent bispecific antibody for T-cell-mediated killing of HER3-expressing cancer cells
Source: Sci Rep. 2021 Jul 6;11:13880. doi: 10.1038/s41598-021-93351-0 (PMC8260734; doi:10.1038/s41598-021-93351-0)
Supplement: Supplementary file 1 — Supplementary Figure 1. [file 41598_2021_93351_MOESM1_ESM.docx]

**Supplementary material**


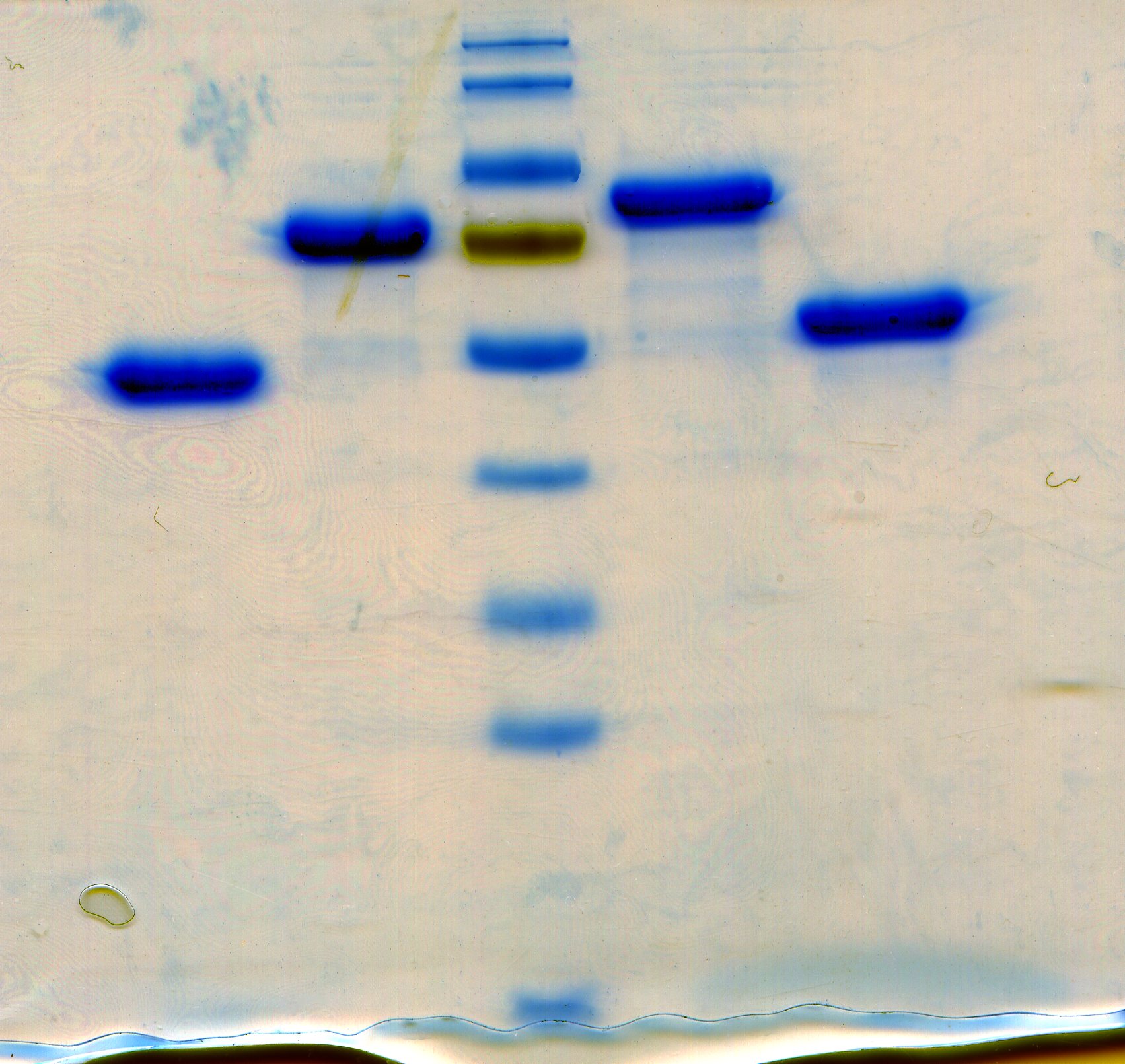


**Suppl. Fig. 1:** Original scan of the coomassie brilliant blue stained SDS-PAGE gel shown in Figure 1b.
